# Managing depression with complementary and alternative medicine therapies: a scientometric analysis and visualization of research activities

**DOI:** 10.3389/fpsyt.2023.1288346

**Published:** 2023-11-15

**Authors:** Fei-Yi Zhao, Peijie Xu, Zhen Zheng, Russell Conduit, Yan Xu, Li-Ping Yue, Hui-Ru Wang, Yan-Mei Wang, Yuan-Xin Li, Chun-Yan Li, Wen-Jing Zhang, Qiang-Qiang Fu, Gerard A. Kennedy

**Affiliations:** ^1^Department of Nursing, School of International Medical Technology, Shanghai Sanda University, Shanghai, China; ^2^Shanghai Municipal Hospital of Traditional Chinese Medicine, Shanghai University of Traditional Chinese Medicine, Shanghai, China; ^3^School of Computing Technologies, RMIT University, Melbourne, VIC, Australia; ^4^School of Health and Biomedical Sciences, RMIT University, Bundoora, VIC, Australia; ^5^Yangpu Hospital, School of Medicine, Tongji University, Shanghai, China; ^6^Institute of Health and Wellbeing, Federation University, Mount Helen, VIC, Australia; ^7^Institute for Breathing and Sleep, Austin Health, Heidelberg, VIC, Australia

**Keywords:** complementary and alternative medicine, depression, depressive disorders, bibliometrics, scientometrics, visualization, VOSviewer, Citespace

## Abstract

**Background:**

Complementary and Alternative Medicine (CAM) interventions may prove to be an attractive option for the treatment of depression. The aim of this scientometric analysis is to determine the global scientific output of research regarding managing depression with CAM and identify the hotspots and frontiers within this theme.

**Methods:**

Publications regarding the utilization of CAM for treating depression were collected from the Web of Science Core Collection from 1993 to 2022, and analyzed and visualized by Bibliometrix *R*-package, VOSviewer, and CiteSpace.

**Results:**

A total of 1,710 publications were acquired. The number of annual publications showed an overall rapid upward trend, with the figure peaking at 179 in 2021. The USA was the leading research center. Totally 2,323 distinct institutions involving 7,638 scholars contributed to the research theme. However, most of the cooperation was limited to within the same country, institution or research team. The *Journal of Alternative and Complementary Medicine* was the most productive periodical. The CAM therapies of most interest to researchers were acupuncture and body–mind techniques, such as yoga, meditation and mindfulness. Systematic review and meta-analysis are commonly used methods. “Inflammation,” “rating scale” and “psychological stress” were identified as the most studied trend topics recently.

**Conclusion:**

Managing depression with evidence-based CAM treatment is gaining attention globally. Body–mind techniques and acupuncture are growing research hotspots or emerging trending topics. Future studies are predicted to potentially investigate the possible mechanisms of action underlying CAM treatments in reducing depression in terms of modulation of psychological stress and inflammation levels. Cross-countries/institutes/team research collaborations should be encouraged and further enhanced.

## 1. Introduction

Depression is a primary mental health condition that contributes significantly to the global burden of disease and affects over 300 million people (1). Depression is responsible for considerable disability and subsequent productivity loss globally, and ranks as the sixth-leading source of high disability adjusted life-years at all ages (2, 3). In comparison to non-mood disorder population, those with depression are 4.5 times more likely to report significant limitations in participating in education, work and household domestics work activities (3). A current unremitting depression is also a strong predictor of all-cause mortality (4), and strongly associated with completed suicide and non-fatal suicidal behaviors (5). Apparently, depression represents an entrenched obstacle to sustainable development in some regions (1). In addition to the increased utilization of public healthcare resources (i.e., increased hospitalizations, hospital days, physician and mental healthcare service provider visits), depression imposes a substantial financial burden on the sufferers and society (6, 7). A model based on combined epidemiological and economic data suggests that the total annual cost of depression in Europe is estimated to be approximately 118 billion EUR (8). This data is estimated at around 4,049 million USD in South Korea (9), and 6,264 million USD in China (10). The COVID-19 pandemic with adverse societal factors including but not limiting to the sickness, bereavement, impoverishment, and social isolation has caused negative impacts on the mental health of millions of people, which likely to further reinforce the need to make the prevention and treatment of depression an urgent worldwide priority (11).

In primary care, depression is most often managed with antidepressants, psychotherapy or a combination of both (12). Antidepressant medication such as selective serotonin reuptake inhibitors are the recommended front-line treatment, yet approximately 30% of patients do not respond to this class of drugs (13). The inability to tolerate the antidepressants’ adverse events, such as dry mouth, sexual dysfunction, weight gain, sleep disturbance and constipation (14) generally results in the decreased adherence and high dropout rates in some patients (13). Despite satisfactory effects (15), psychotherapeutic treatment as a restricted resource is usually undersupplied and associated with prolonged waiting periods, particularly in rural areas (16). Similar to pharmacotherapy, premature discontinuation is also a widespread problem amongst patients treated with psychotherapy (17, 18). Such early cessation of pharmacological and/or psychotherapy leads to poorer clinical outcomes and negatively impacts the best allocation of scarce clinical resources (18).

Complementary and alternative medicine (CAM) is also popularly taken up by patients who are depressed (12, 19). CAM refers to a diverse range of products and practices used in the management of health conditions but typically not part of the dominant health care systems (20). CAM treatment involves five categories with multiple modalities relying on their primary therapeutic input, namely nutritional, physical, psychological, combinations such as physical and psychological or psychological and nutritional therapies, and other complementary health approaches (Appendix 1) (21). Globally, depression is one of the ten most leading indications for seeking CAM treatment (22). More than one-third of Americans utilize CAM therapies in a given year. Furthermore, the use of CAM is more common among individuals with psychiatric complaints than the rest of the population (19). A survey covering 6,618 adults in Australia indicated that self-help strategies including CAM were very commonly adopted to cope with depression, with meditation, music therapy, and massage often being used to address mild depression, and *St John’s Wort*, aromatherapy, nutritional supplements and yoga often being used to address moderate depression (23).

Over the past few decades, the use of CAM has grown in popularity and orthodox healthcare providers have acknowledged the value of the existence of CAM. This is attributed to the surge in CAM research productivity, that is the increase in the amount of published literature available that details therapies (24). The identification of the dynamic evolution of disciplinary development from extremely large corpora of associated research papers, however, represents a considerable challenge to scientific scholars (25). The scientometric analysis is a powerful quantitative approach to analyze the distribution structure, quantitative relationship and change rules of documents, using mathematics, statistics and other estimation and measurement techniques (26). Compared with a traditional narrative review by experts, which usually subjectively focus on the progress in a specific research field, scientometric analysis is advantageous in objectively, comprehensively, and quantitatively summarizing the whole topic based on the information best available (27, 28). Featured by comprehensive literature search and critical assessment, a systematic review is more rigorous and may provide less biased evidence than a narrative review, and it can be either qualitative or quantitative (meta-analysis) (29, 30). It has been suggested that systematic reviews attempt to answer a specific research question based on a small number of publications that meet strict inclusion criteria for homogeneity, whereas scientometric analysis aims to answer a specific research question based on a large number of publications (31). Scientometric analysis also differs from a scoping review. A scoping review is designed to determine the type and scope of research evidence, whereas scientometric analysis gives a valuable overview of a field’s national and worldwide contributions to literature (31) by both summarizing historical research achievements and predicting future research trends (25). More importantly, unlike literature reviews, the results of scientometric analysis are intuitive and accessible through being displayed with visualization techniques (27). Such advantages facilitate scholars to gain a one-stop overview, identify knowledge gaps, and explore the intellectual structure of a specific domain (27). All of these strengths scientometric analysis immensely popular in academic circles in recent years (25).

Many narrative (32, 33) and systematic reviews (34), as well as meta-analyses (12) regarding the utilization of CAM for the management of depression have been published. However, as aforementioned, literature reviews relying on qualitative techniques could be marred by interpretation bias from scholars across different academic backgrounds (28). Although meta-analysis is a quantitative method, it serves a different research purpose than bibliometrics. Meta-analysis is used to summarize evidence by analyzing the direction and strength of effects and relationships among variables, while scientometric analysis is used to identify the intellectual structure of a specific field by analyzing the social and structural relationships between different research constituents (e.g., countries/regions, institutions, authors, etc.) (28). In addition, previously published reviews have provided the evidence on efficacy and safety/risks of CAM in the management of depression, as well as the quality of that evidence, but did not include other critical results such as global dynamic development trends, achievements, trends and cumulative patterns of publications, and other keystone bibliometric indices in the field of managing depression with CAM. However, these outcomes are significant in shedding light on the research status, research hotspots, as well as research trends and frontiers within this research field (35). A bibliometric analysis on the current research topic therefore is warranted.

We are aware that there have been other bibliometric studies focusing on specific CAM therapies, such as aromatherapy (36), acupuncture (37), yoga and *Yagya* (38) in treating depression. In contrast, CAM is viewed as an umbrella concept in our current study, enabling a broader perspective for scrutinizing the development trends of overall CAM field. Such scientometric analysis viewing CAM as an umbrella concept may also help to identify which CAM modality has attracted or is attracting the most widespread attention, and which modality is most likely to be the hotspot for future research.

## 2. Methods

### 2.1. Data acquisition and search strategy

This study was carried out with reference to a bibliometric analysis guideline published in 2021 (28). Scientometric analysis relies on literature databases. Providing overall data sources for scientometric software, Web of Science (WoS) is the database most commonly used for such type of research (39). AlRyalat et al. compared bibliometric analysis using Scopus, WoS and PubMed, and confirmed that WoS is the most user-friendly and straight forward database to use for scientometric services (40). The data used in our bibliometric analysis were retrieved from the WoS Core Collection (WoSCC). The search was performed on July 30, 2023, by combining search terms (Appendix 2) from two categories, namely CAM and depression. In the current study, we aimed to gain a macro view of the CAM field, rather than understand the development trend of a specific product or practice in managing depression. We therefore adopted CAM as a broad umbrella term, and used related words as search terms for retrieval, rather than searching each specific type of CAM therapy (Appendix 3). Considering the original contribution and summary of cardinal research findings, only “Article” and “Review” were considered. The less representative record types such as conference abstracts and proceedings, corrigendum documents, editorial material, etc. were filtered out. Papers regarding CAM for depression published in the past 30 years (from 1993 to 2022) in English were retrieved and we then checked the relevance of the results.

We also collected the Journal Citation Reports (JCR) quartile and impact factor (*IF*) of journals, and the *h*-index of scholars from the WoS. The quartile in JCR and *IF* are widely used as two measures of the prestige of a journal (41), and the *h*-index is used to measure the research productivity, impact and quality of a scholar (42, 43).

### 2.2. Bibliometrics and visualization analysis tool and process

To ensure the reliability of the results, two researchers (FY-Z and LP-Y) independently examined the qualifying papers. The full records of the resultant publications were then imported to Bibliometrix package 4.1.2 in *R* Studio 4.2.3, VOSviewer 1.6.8, and Citespce 6.2R4 and for further bibliometric analyses and visualization. Data for analysis, such as countries/regions, affiliations, authors, journals, keywords, and critical indicators for measuring research performance [i.e., number of publications (*Np*), number of citations (*Nc*), and *h*-index] were extracted by the software. The *Np* and *Nc* are usually used to quantify productive capacity and demonstrate impact, respectively (44); and the *h*-index unites productivity and influence by identifying a threshold that links *Np* and *Nc* (44).

Bibliometrix is designed to assist researchers in conducting automated science mapping (45). It supports a recommended workflow to perform bibliometric analyses (45). As Bibliometrix is programmed within *R* environment, it is highly flexible and can be rapidly upgraded and integrated with other *R* packages to support statistical operations (45, 46).

VOSviewer is a literature knowledge visualization software for constructing bibliometric networks (47). In the VOSviewer output, different nodes represent different elements such as countries, regions, institutions, authors, or terms. The links between nodes represent associations, such as co-authorship, co-citation or co-occurrence, and weighted by total link strength (42). The distance between the nodes reflects the degree of relatedness of the nodes (46). The importance of a node in the network was quantitatively determined by its total link strength with other nodes (48). The weight acquired by each node, shown by the number of publications or citations, determines the size of the node in the output (46).

CiteSpace is another bibliometric software and is designed for progressive knowledge domain identification of critical points in the development of a specific field, particularly intellectual turning points and pivotal points (49, 50). It excels at capturing keywords related to strong citation bursts and investigating keywords’ time co-occurrence to predict research frontiers and explore keywords’ co-evolutionary pathways (42). Therefore, we used CiteSpace, in the current study, to make up for the gaps of VOSviewer.

## 3. Results

**Figure 1:**
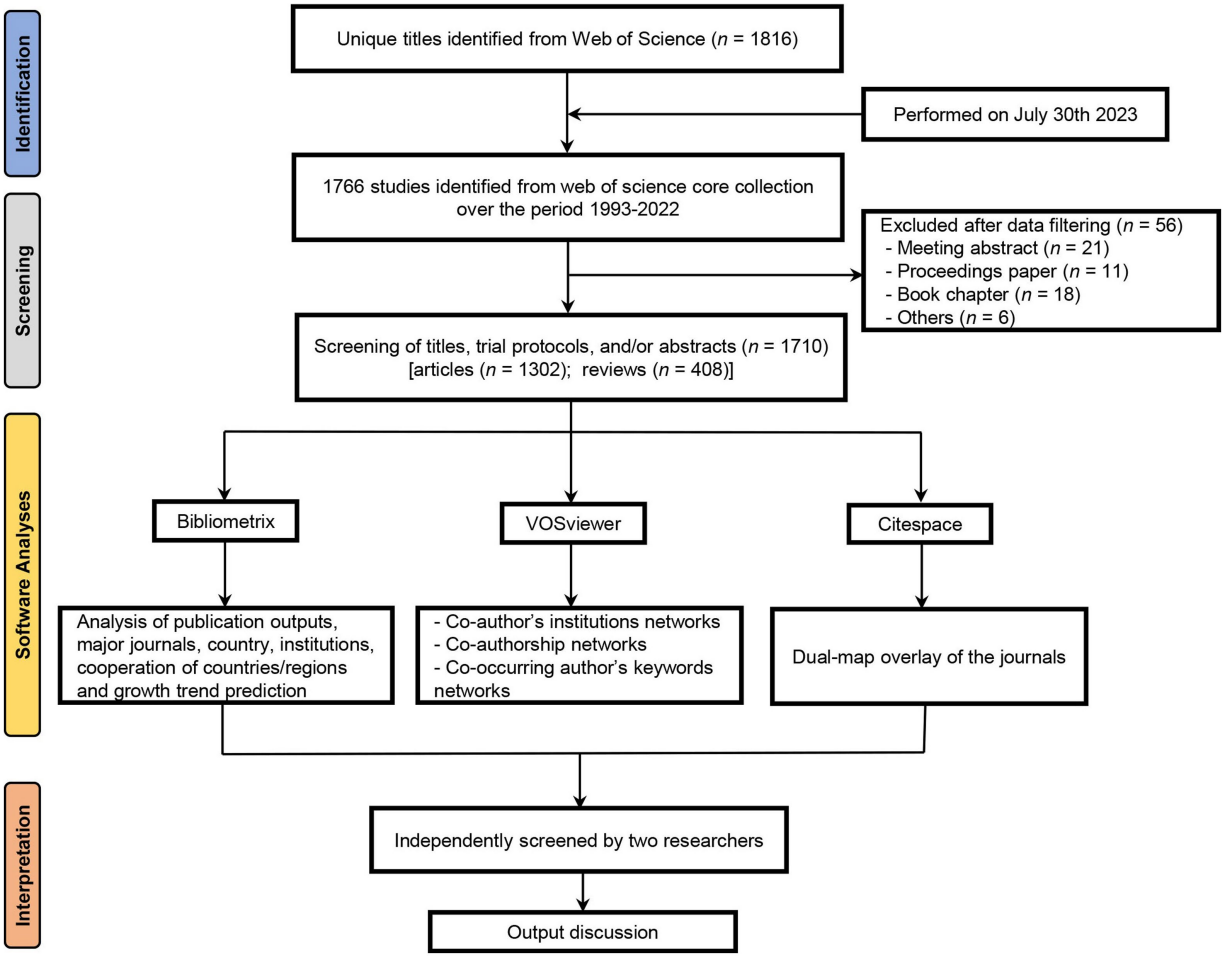
Flow diagram of literature identification.

We identified 1,816 records from the database and finally included 1,710 eligible papers (Figure 1). Of these papers, “Article” was the most dominant publication type (76.1%, 1,302/1,710). The total *Nc* of the retrieved papers was 46,876, and the average *Nc* per paper was 27.40.

### 3.1. Annual publications and trends

**Figure 2:**
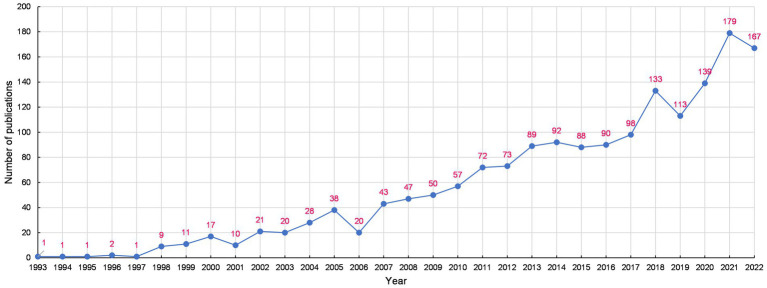
Global trends of annul publications regarding managing depression with CAM from 1993 to 2022.

As revealed in Figure 2, though there was a slight fluctuation in the number of publications regarding managing depression with CAM, it showed an overall annual upward tendency over the past three decades. Among the 1,710 papers included, the first paper was published in 1993. Up to 2001, the number of annual publications did not exceed 20. From 2002 onward, the number of annual publications has shown an overall rapid upward trend year by year, suggesting that the research topic began to enter a period of rapid development. Research outputs peaked in 2021, with the number of annual publications at 179.

### 3.2. Contributions of countries and regions

**Figure 3:**
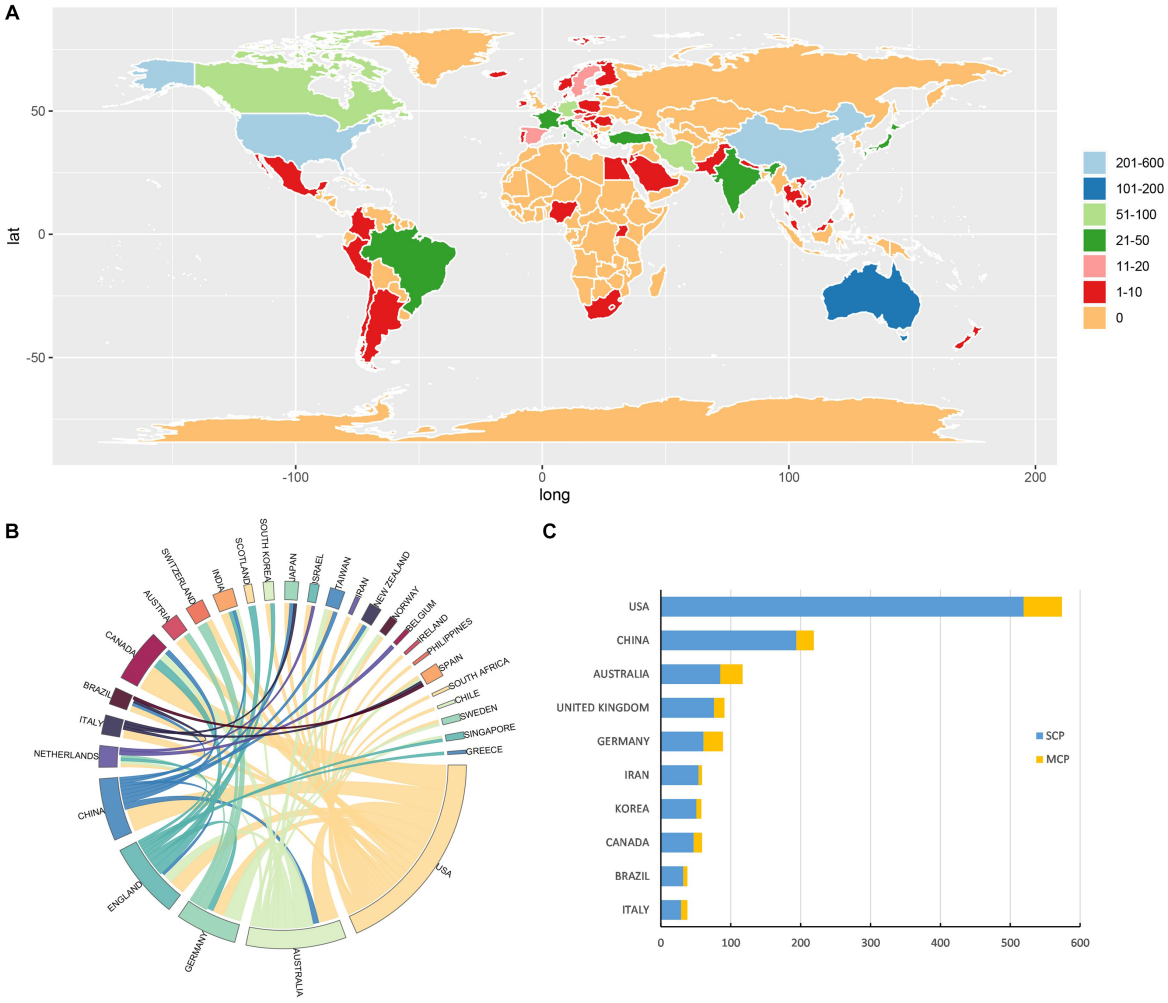
Contribution of countries/regions to the research regarding managing depression with CAM. **(A)** World map displaying the global distribution of current research theme. Different countries are indicated by different colors based on the number of papers published. **(B)** Distribution and international cooperation of countries/regions involved in the current research field. The thickness of the line reflects the frequency of the cooperation; the thicker the line, the closer the cooperation. **(C)** The total number of publications counts and average citation per item of the top 10 most productive countries that contributed research within the current focused field. SCP, single country publications; MCP, multiple country publications.

There were 68 countries/regions that published research regarding using CAM therapies in the management of depression (Figure 3A). Among these countries/regions, there are 29 countries/regions where the total link strength between two countries/regions was equal to or greater than three, and were included for country/region co-authorship analysis. Notably, the USA was at the center of research in this field with relatively close collaborations with Australia, China and Canada. Germany and Switzerland also consistently worked together on research related to CAM management for depression. Despite relatively low research output, the lines’ strength and count of some countries/regions such as Netherlands, Italy and Brazil indicated consistent connections with other countries/regions and might have a potential impact on other countries/regions’ research (Figure 3B). However, numbers of collaborative research outputs between countries are much less than that of independent research outputs in their own countries (Figure 3C).

We also rated the top ten high-output countries/regions. The USA was the most productive and impactful country/region, with total 574 papers published, followed by the China (219/12.8%) and Australia (117/6.8%). Amongst these 10 countries, the countries with the most research cooperation with other countries were, in order, the USA, China and Australia. Italy, although lower than other countries in number of publications, was higher than Iran, South Korea and Brazil in the closeness of research cooperation with other countries (Figure 3C; Appendix 4).

The USA was also the country/region where papers were most cited, with papers referenced 24,205 times. Although there were less papers published in Brazil than in Iran and South Korea, the total citations for papers published in Brazil was much higher than citations for papers published in the other two countries (Appendix 4).

### 3.3. Contributions of institutions

**Figure 4:**
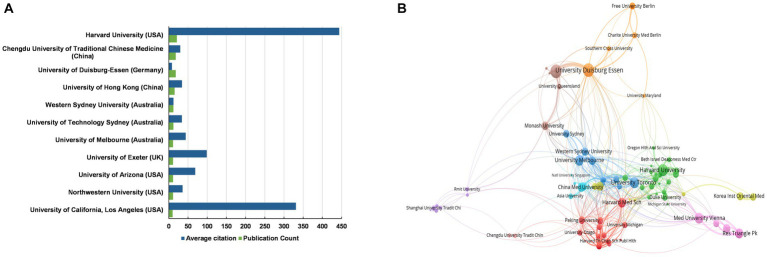
Contribution of institutions on research regarding managing depression with CAM. **(A)** The total number of publications counts and average citation per item of institutions that contributed a minimum of 10 papers in the current research field. **(B)** Mapping of the co-authorship analysis among top 100 most productive institutions in the current research field. Each node represents an institution, and the node size indicates the number of publications. The connection between the nodes represents a co-authorship relationship, and the thickness of the lines indicates strength (weights on the total link strength).

Totally 2,323 distinct affiliations were involved in the publication of current research theme. Among them, 979 institutions published papers as the corresponding author affiliation. In these 979 institutions, 11 institutions published a minimum of 10 papers. Most of these 11 institutions were located in the USA, Australia or China, which is consistent with the findings derived from the contributions of countries/regions. Evidently, these institutions play a crucial role in expanding national influence. Harvard University (USA) was found to be the most productive institution with 21 papers, followed by the University of Duisburg-Essen (Germany) and the Chengdu University of Traditional Chinese Medicine (China) each with 18 papers. On average, papers from Harvard University (USA) were cited 444.1 times, which was much higher than the number of citations for other institutions (Figure 4A; Appendix 5).

In addition, 747 links were found among the top 100 most productive institutions in the co-author analysis network (Figure 4B).

### 3.4. Contributions of authors

A total of 7,638 authors contributed to the research regarding CAM therapies for depression, and 11 authors published at least 10 papers. The top ten most productive authors were listed in Table 1. They published 147 publications, accounting for 8.6% of all papers submitted. Jon Adams from University of Technology Sydney (Australia) was the most productive and impactful author in this field with 21 papers published, followed by Holger Crame (19 papers) from University of Tuebingen (Germany) and Edzard Ernst (16 papers) from University of Exeter (UK). Roger B Davis’s research work had an extremely high impact, with the highest number of citations at 5,107. Edzard Ernst’s research work has attracted more scholars’ attention, with the highest *h*-index at 13. Furthermore, among the top 10 authors, Edzard Ernst was also the first scholar to publish a paper in the research field. We notice that the top 10 authors were mainly from the USA, Australia and Germany. It suggests that there are more excellent researchers focusing on CAM management of depression in these three countries.

**Table 1:** The top 10 authors with the highest productivity.

Rank	Author	Affiliations	Country	*PSY*	*Np*	*Nc*	*h*-index	*g*-index	*m*-index
1	Jon Adams	University of Technology Sydney	Australia	2008	21	299	11	17	0.688
2	Holger Cramer	University of Tuebingen	Germany	2012	19	316	8	16	0.667
3	Edzard Ernst	University of Exeter	UK	1998	16	1,074	13	16	0.500
4	David Sibbritt	University of Technology Sydney	Australia	2008	16	238	10	15	0.625
5	Jost Langhorst	University of Duisburg-Essen	Germany	2011	15	380	9	12	0.692
6	Romy Lauche	Southern Cross University	Australia	2012	15	270	7	11	0.583
7	Roger B. Davis	Harvard Medical School	USA	1998	12	5,107	9	12	0.346
8	Gustav J. Dobos	University of Duisburg-Essen	Germany	2011	12	174	7	10	0.538
9	David Mischoulon	Harvard Medical School	USA	2010	11	289	8	11	0.571
10	Paula Gardiner	University of Massachusetts	USA	2013	10	116	7	8	0.636

Np, number of publications; Nc, number of citations; PSY, publication start year.

**Figure 5:**
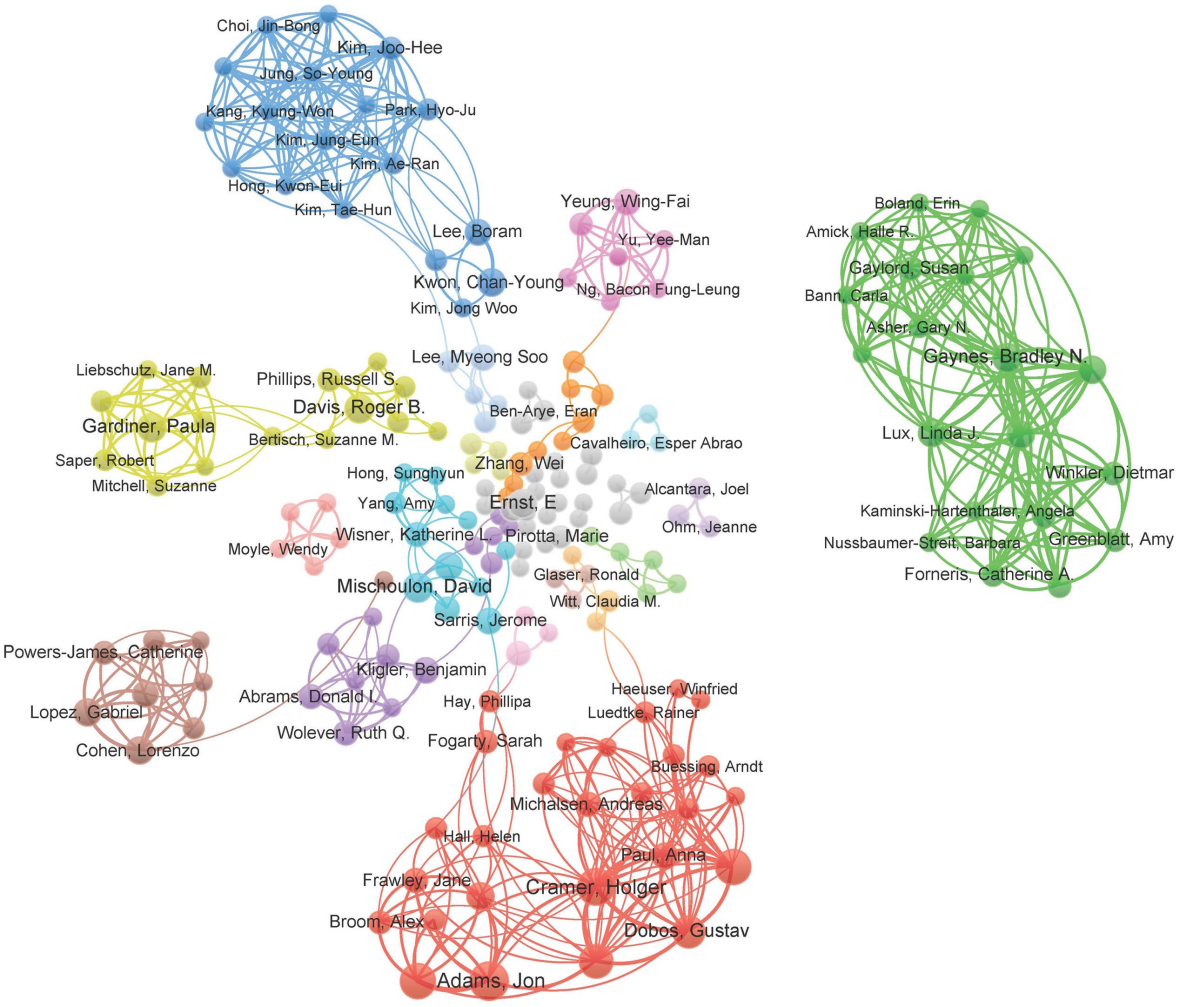
Mapping of the co-authorship analysis among the authors who published at least three papers on research regarding managing depression with CAM. Each node represents an institution, and the node size indicates the number of publications. The connection between the nodes represents a co-authorship relationship, and the thickness of the lines indicates strength (weights on the total link strength).

In accordance with the *Price’s Law* (51), *m* = 0.749 X $\sqrt{\max\left(n\right)}$ (where *m* represents the minimum number of papers published by core authors, *max(n)* represents the number of papers by the author with the most publications in the field), authors who have published equal to or more than three papers were determined as the core authors in the current research field. A total of 219 authors (publications ≥3) were included in a network map of authors and were grouped into nine clusters (Figure 5). Active collaborations usually exist in the same cluster, such as Rainer Luedtke and Gustav J Dobos. There were also collaborations among linked two nodes in different clusters, such as Rainer Luedtke and Claudia M Witt.

### 3.5. Journal analysis

Collectively, 658 academic journals published articles on research regarding CAM therapies for depression. The top 10 most productive journals published 337 papers on this theme, accounting for 19.7% of all publications (Appendix 6). *Journal of Alternative and Complementary Medicine* had the greatest number of publications (*n* = 61), followed by *Complementary Therapies in Medicine* (*n* = 41) and *Evidence-Based Complementary and Alternative Medicine* (*n* = 40). The journal with the highest *Nc* (*n* = 5,391) was *JAMA - Journal of the American Medical Association* (*IF*: 120.7), although it only published five papers within the current topic. Half of the top 10 active journals were in the CAM field, and the remaining journals were comprehensive medical journals, or journals in the fields of psychiatry, psychology, or oncology. According to the JCR 2022 standards, most of these productive journals ranged from Q3 to Q1. We note that *Evidence-Based Complementary and Alternative Medicine* (JCR Q3 and *IF*: 2.650 in 2021–2022) was moved out of the SCIE index in the 2023 update to its annual Journal Citation Reports.

**Figure 6:**
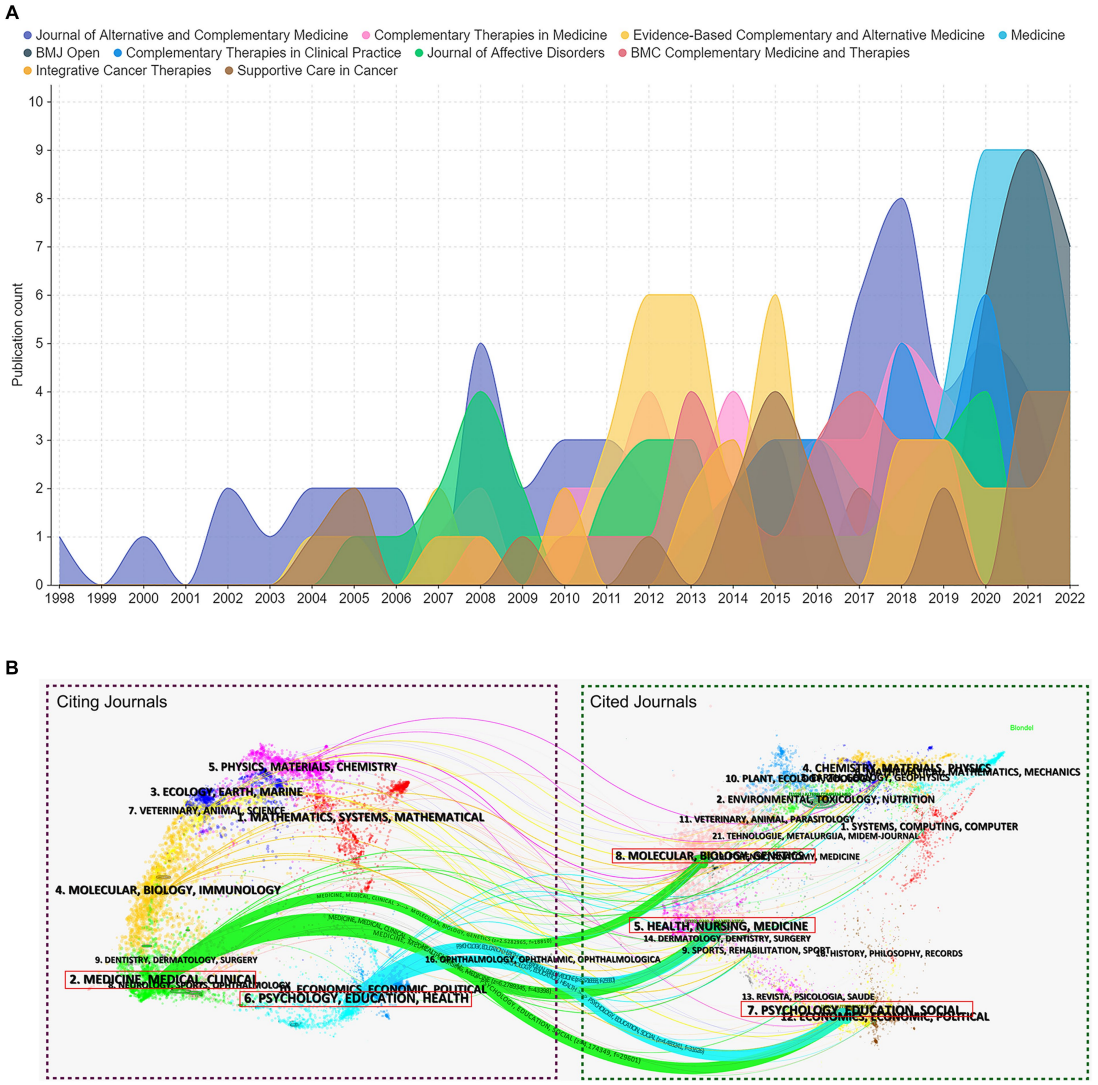
Contribution of journals in the research field of managing depression with CAM. **(A)** Growth trends in the publication quantity of the top 10 productive journals in the current research theme from 1993 to 2022. **(B)** Dual-map overlay of the journals on the current research theme. The labels represent different research subjects covered by the journals. The citing journals are on the left side, while the other side of the map represents the cited journals. Different colored lines correspond to the different paths of references, beginning with the citing map and ending at the cited map. The main citing and cited journals are shown in the red box.

*Journal of Alternative and Complementary Medicine* has been publishing papers regarding CAM therapies for depression since 1998 and continues to do so today. The peak in the number of articles published on this topic, in this journal, occurred in 2018. After that, it has lost its numerical dominance in publishing papers on this topic to two comprehensive medical journals, i.e., *Medicine* and *BMJ Open*. Although *Supportive Care in Cancer* and *Integrative Cancer Therapies* are not journals in CAM field, they published papers related to the current research topic from 2004 and 2007, respectively (Figure 6A).

Figure 6B is a dual-map overlay, showing the co-occurrence network regarding CAM therapies for depression. Overall, there were five major citation paths on the current map. The citing matrices of journals were mainly in two fields: (1) medicine, medical, and clinical, and (2) psychology, education, and health, whereas the most cited publications originated from the journals in the field of (1) health, nursing, and medicine, (2) psychology, education, and social, and (3) molecular biology and genetics.

### 3.6. Keyword analysis of trending research topic

**Figure 7:**
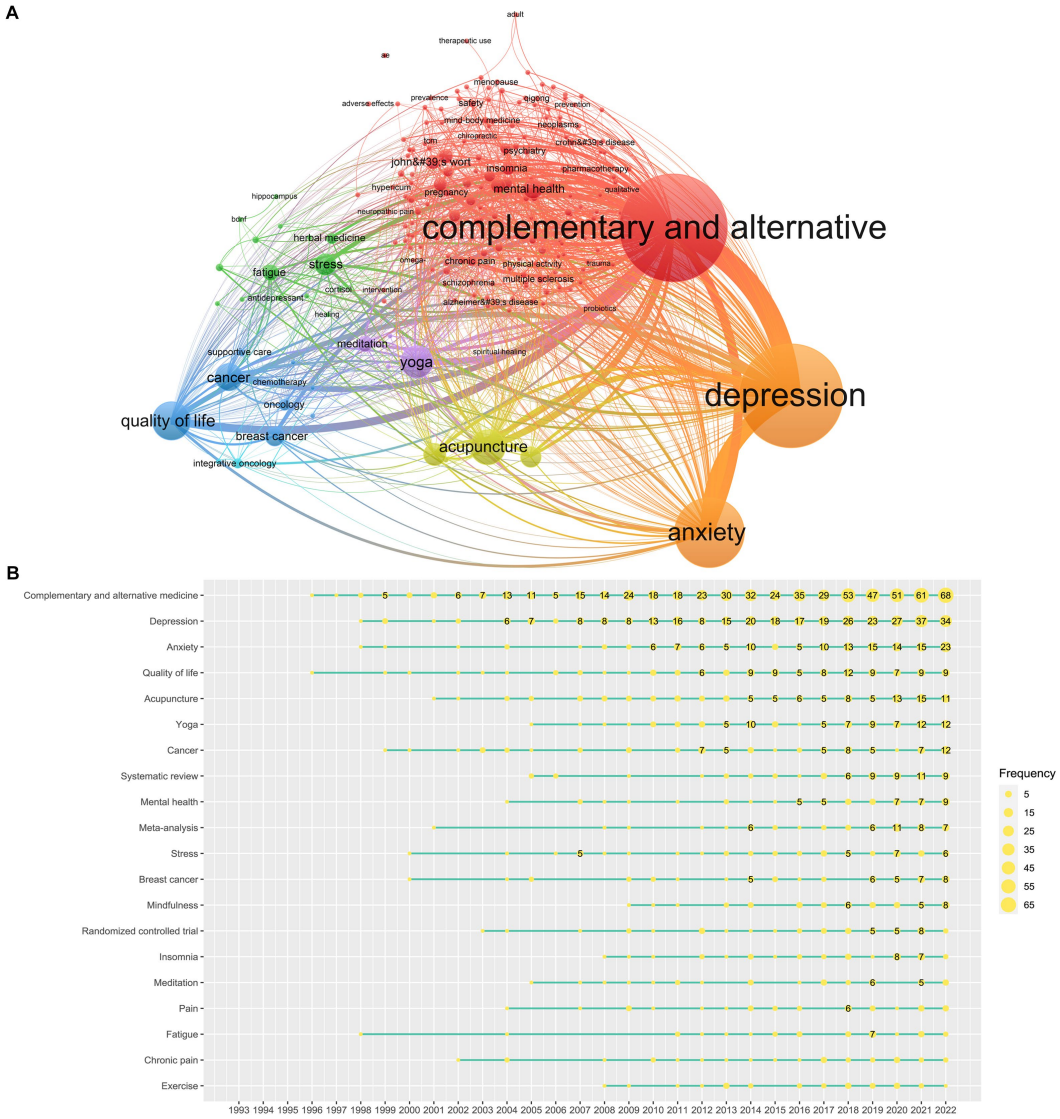
Mapping of keywords in the research field regarding managing depression with CAM. **(A)** Keyword co-occurrence analysis on the current research theme. **(B)** Keyword time zone on the current research theme over the years.

To trace the developing trends and hot topics in the research field of CAM therapies for depression, we performed a keyword co-occurrence analysis using VOSviewer. A total of 2,950 keywords in the 1,710 publications were identified. Among them, 219 keywords with frequency higher than five were included in the co-occurrence network and majorly divided into six clusters (Figure 7A). The keywords clustered in the red region included the main terms associated with CAM treatments. Keywords clustered in the green region mainly described the topic of depression and anxiety. Depression is the subject of our current research, and anxiety are often presented as a concomitant/comorbid symptom of depressive disorder. The keywords clustered in the yellow and purple regions displayed some common CAM therapies used for depression, such as acupuncture, yoga, and meditation. The keywords clustered in the blue region were mainly associated with cancer and quality of life, implying that many studies might have focused on the use of CAM in cancer patients with depression symptoms. The keywords clustered in the green region focused on etiology and mechanisms.

To understand the evolution of new hotspots, we further analyzed the 20 most frequent keywords using the keywordGrowth function in *R*. As shown in the keyword time zone view, systematic review and meta-analysis have been two popular research methods in the last decade. Among CAM modalities, acupuncture and yoga appeared to have received the most attention. In addition, cancer, especially breast cancer, has been a high-frequency keyword with respect to the current research topic (Figure 7B).

**Figure 8:**
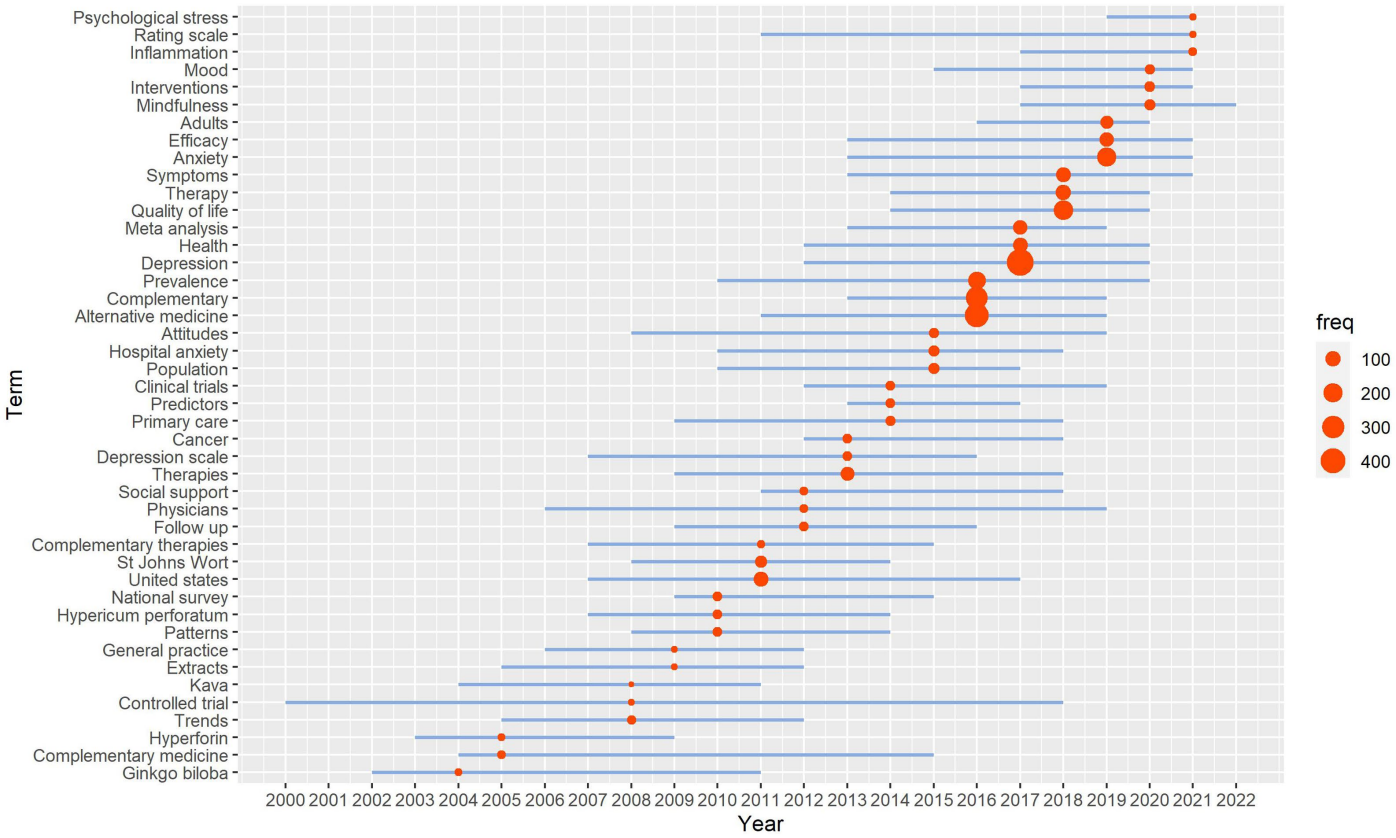
Trend topics in research regarding CAM therapies for depression over the years.

We also selected keywords that appeared at least five times over 3 years to characterize the emerging topics in the target discipline, and in turn predict future research trends (Figure 8; Appendix 7). The most studied trend topics over the years (1993–2022) were mainly related to CAM, depression, prevalence, quality of life, symptoms, research methods (clinical trials and national-survey), phytomedicine (*Kava*, *St John’s wort*, and *Ginkgo biloba*). However, inflammation, rating scale, psychological stress, and mindfulness were the most studied trend topics recently and might become the future research hotspots.

## 4. Discussion

### 4.1. Summary of findings

The current study adopted a quantitative scientometric method, and provides a comprehensive snapshot concerning CAM treatment for depression over the previous 30 years. The relevant publications in general show a rapid year-on-year increase trend, showing this research theme is gaining attention worldwide. The USA, China, and Australia were the main research powers in the current research field. This may be due to the fact that more affiliations or excellent scholars in these countries are looking at the target research, thus expanding the national influence in the field. Harvard University was the institution with the highest research strength and a strong citation burst, with published papers were cited 444.1 times on average. Roger B. Davis from this institution was one of the most influential authors. However, collaboration among authors within the current research field was less than intimate and it was usually limited to one institution or one country/region. Cross-team cooperation was insufficient. The studies on the management of depression using CAM involve multidisciplinary fields, including psychology, clinical science, nursing, sociology, education, and molecular biology. In addition to being a stand-alone mental illness, when depression is viewed as a comorbidity in cancer patients, CAM treatments for this symptom have widely received attention. Of the various CAM modalities, researchers are most keen to study mind–body techniques and acupuncture, with the research focus gradually shifted from effects and safety assessment to investigation of mechanisms of actions.

### 4.2. Strengths, limitations, and comparison with previous scientometric analysis

In comparison with the previously published various types of reviews within the similar topic, our current scientometric analysis provides a more timely, visual, and unbiased approach to track the emerging trends and frontiers (25), and outlines the intellectual structure of a knowledge domain regarding CAM use in the management of depression. We are aware of a bibliometric analysis published in 2022 that investigated trends in CAM treatment for common mental disorders from 2001 to 2020 (35). That article differs from our study in at least two areas. First, that article did not focus on a specific mental disorder, as a result, there was limited findings about depression. Second, the bibliometric indicators reported in that article only included time trend, country and organizations contribution, co-authors and keywords (35). Our current study had additional data of journal analysis and future research prediction based on characterizing the emerging topics. Journal analysis identified the citing/cited matrices within the target topic not limited to clinical medicine and nursing, but also involved in psychology, education and social science, as well as molecular biology and genetics (Figure 6B). Highly compatible with this result, one predicted promising research direction was clarifying the possible mechanisms underlying anti-depressive effects of CAM therapies in terms of modulation of psychological stress and neural inflammation (Figure 8). The quality of this study is further augmented by the diversified academic background of the researchers and our interactive multidisciplinary collaboration. The disciplinary backgrounds of the research team members span psychiatry, nursing, clinical psychology, CAM, public health as well as computer science and information technology, covering all aspects of the present research topic and methodologies adopted.

Few limitations should be acknowledged. First, nearly all information was extracted and analyzed by bibliometric software based on machine learning and natural language processing, which may lead to potential bias (52). Second, due to the continuous updating of databases, the scientometric analysis results might fall behind the actual research status (48).

### 4.3. Interpretation of the findings

The continued growth in the number of publications implies that more researchers are becoming interested in managing depression with CAM. The ICD-11 code set, which was officially in effect in 2022, first contains traditional medicine diagnoses, recognizing the value of traditional medicine in disease management and health promotion (53). Member states of WHO are also encouraged to integrate such practices into their healthcare systems in addition to regulations and research (53). Therefore, we predict that the increasing trend of publications on current research theme is likely to continue in the years to come.

Instead of being marginalized, some CAM systems, such as traditional Chinese medicine (54) and traditional Korean medicine (55), are regarded as “mainstream medical practices” alongside Western medicine in their own countries. Interestingly, according to results, the USA, rather than these countries that place a high value on CAM, was the most active and highly contributing country in the current research theme. Four institutions and three scholars of the USA were in the top 10 affiliations and authors in the research field (Appendix 5; Table 1). This is in line with the result derived from previously published bibliometric studies conducted in other research fields (48, 56, 57). One possible explanation for this is that the USA allocates a large budget for research, science and technology to support more exceptional institutions and specialist scholars carry out active research activities, and strongly collaborated with institutions/researchers from other developed countries (57). The National Center for CAM (NCCAM) experience in the USA has also demonstrated that when funds are available and priorities are set, CAM research will grow exponentially (58).

Although 7,638 scholars contributed to the current research theme, most of the cooperation between them is limited to the same country or same institution. Such forms of collaboration with close internal connection but less external connection restricts the sharing of resources, ideas, and the exchange and discussion of individual perspectives among researchers (26). To produce more high-quality outputs in the current research field, cross-team (i.e., cross-institutional and cross-regional) collaboration is warranted. We also noticed that the authors who were located at a central position of the cooperating clusters in the co-authorship analysis were majorly from the affiliations in the United States and Europe rather than Eastern countries where CAM is more prosperous. This may be associated with the language barrier, which is considered part of the blocks of international collaboration (48). It is gratifying that a few Korean and Chinese authors were actively involved in the cooperating clusters, and their significant academic share are hoped can encourage more researchers to devote themselves to this field.

Most of the papers regarding CAM treatment for depression were published in the CAM journals. After all, the scientific directions covered by these journals are more relevant to the current research topic, thus being more likely to encourage scholars to submit their works to these journals (44). However, the *IF* of all these journals is below 4.0, indicating that the academic value and impact of such papers still need to be strengthened. It has been suggested that CAM-related papers were rarely accepted by mainstream medical journals, particularly those with high *IF*. This is because many clinical trials (and their resulting reports) of CAM therapies were of poor methodological quality (59). Part of the reason for the poor quality is that conducting CAM trial is complex and has its own unique challenges and difficulties. For instance, finding appropriate placebos or shams for treatments such as chiropractic, massage/Tuina therapy, or complex herbal mixtures is challenging; double blinding of some interventions such as acupuncture and massage may not be possible (60). Therefore, it may be biased to criticize CAM trials for methodological quality flaws using the methodology developed to appraise typical pharmaceutical drug trials. Reputable research institutions such as U.S. National Institutes of Health have recognized this problem and have called for appropriate research paradigms to be designed for CAM trial to achieve high-quality evidence, despite increases in complexity and possibly cost associated with such designs (60). Our scientometric analysis is consistent with the general trend that a small number of studies regarding managing depression with CAM have been published in the influential comprehensive medical journals or psychiatric periodicals, besides CAM periodicals. Despite being challenging, we still suggest that outstanding and original discoveries should be considered to be submitted to mainstream medical journals to gain more discourse regarding managing depression with CAM in the orthodox medicine field. Those periodicals with a high degree of popularity and impact can also facilitate CAM academics promote their ideals or opinion in the field of science, allowing them to discuss and exchange their ideas with mainstream medicine peers in order to improve their academic level and science ability (44). In fact, many clinical practice guidelines regarding depression treatment compiled by the orthodox medical societies included CAM-related recommendations (61). This is also in line with the WHO’s vision of future health service delivery, i.e., it is efficient blend of both traditional and conventional medicine where practitioners and patients work together on disease prevention and health promotion (53). However, CAM therapy will only be recommended by guidelines if there is reliable evidence of its effectiveness and safety. The development of clinical guidelines often relies heavily on systematic reviews to directly provide clear and reliable evidence (62). Coincidentally, keyword time zone view identified that systematic review and meta-analysis were two popular research methods in the last decade (Figure 7B). Apparently, CAM researchers have also been aware of the need to critically review the existing evidence within the current research topic with evidence-based principles.

In addition to being a stand-alone mental disorder, depression is a common comorbidity amongst cancer survivors. An emerging systematic reviewed revealed that the global prevalence of depression in male and female cancer patients was 26 and 31%, respectively. Furthermore, this rate is increasing by an average of 0.6% per year (63). Amongst breast cancer patients, the prevalence of depression was around 32.2% (64). Cluster analysis and burst disclosed the research hotspots and frontiers in the field of managing depression with CAM. Many terms associated with tumor, such as oncology, integrative oncology, cancer, breast cancer chemotherapy, and supportive care were identified as the high-frequency keywords (Figure 7A). Evidently, the use of CAM therapies to manage depression in cancer survivors has attracted the attention of researchers. The journal analysis also supports the current findings. Two of the top 10 journals in terms of publications are specialized oncology treatment/care journals (Appendix 6).

Capturing the growing research hotspots helps countries, scholars and policymakers better understand the field and make effective decisions (44). Co-occurrence analysis revealed that yoga and acupuncture are two CAM modalities of wide interest to researchers in the management of depression (Figure 7A). A large number of systematic reviews and meta-analysis have been published concerning yoga treatment for depression. All these reviews indicated the positive benefits of yoga (65–67). Geng et al. compared the effects of several mind–body exercises on depression among breast cancer survivors (another hotspot identified in this study) using the network meta-analysis. They reported that yoga was more effective than Baduanjin, Pilates, dance, and Qigong (68). There are also several systematic reviews and meta-analyses on acupuncture, indicating that acupuncture might have some benefits in ameliorating depression, either being used alone or being used in combination with conventional treatments (69–71). Systematic reviews of both yoga and acupuncture, however, have emphasized that the positive results of yoga should be interpreted with caution due to the high risk of bias in current trials (65–67, 69–71). However, as we cautioned earlier, it may be biased to criticize CAM trials using drug trial method, particularly “gold standard” double-blind placebo randomized controlled trials (RCTs) (72). There is even a misconception that all RCTs require to be blinded and that interventions within an RCT cannot be individualized (73). The fact remains, however, that CAM therapies are usually very individualistic in approach and cannot always be standardized as a treatment for large groups of individuals in the context of an RCT (72), nor some therapies are possible to blind (74). RCTs also attempt to “control out” non-specific effect which in many CAM modalities appears to play a critical role in producing a positive outcome (72). Hence, for promising CAM therapies, including yoga and acupuncture, it is recommended that their clinical value be determined through more appropriate research paradigms and evidence evaluation methods. Single case designs, qualitative approaches, outcome assessment and clinical audit, and observational studies have been suggested, as alternative research methods, to be used in examining the effectiveness of CAM therapies (72). In addition to the investigation on efficacy and safety, biomedical and medical imaging techniques are suggested to disclose the mechanisms underlying yoga and acupuncture. Halappa et al. found that stress reduction, by way of inhibiting the overactivated HPA axis, was particularly relevant to the effect of yoga in alleviating depression (75). Several trials by Quah-Smith’s team showed that laser acupuncture had a clinically and statistically significant benefit in reducing symptoms of depression and it was well tolerated with transient fatigue being the only adverse effect (76–78). Using functional magnetic resonance imaging (fMRI), it was found that the antidepressive effect of laser acupuncture involved modulation of the default mode network (DMN), particularly the wider posterior DMN modulation of the parieto-temporal-limbic cortices (79).

Besides overviewing historical trends and current research status, scientometric analysis provides clues to future promising research directions (26). Inflammation, psychological stress and mindfulness were identified as potential research priorities in the future (Figure 8). There is already strong evidence that inflammation plays a role in the pathophysiology of depression, i.e., it can contribute to the development and progression of depression by altering brain chemistry (80). Similarly, psychological stress is usually viewed as a trigger of depression onset (81). Individuals who are vulnerable to depression tend to develop negative bias under mild psychological stress (82). Effects of stress on the regulation of inflammatory and immune processes also have the potential to influence depression (81). We therefore predict that future research may focus on the mechanisms of action underlying CAM therapies to ameliorate depression, i.e., whether such antidepressive effect is achieved by modulating the inflammation levels and/or psychological stress states. In fact, there have already been some studies in this field. A fMRI study with psychometric testing found that mindfulness might reduce vulnerability to depression by reducing automatic emotional responding via the insula as well as buffering against trait rumination and negative bias (82). More evidence is with respect to the inflammation-mediated hypothesis. Based on the chronic unpredictable stress rat model, Lu et al. proved that the antidepressant-like effect of acupuncture was mediated by inhibition of inflammatory mediators via modulation of NF-κB in the brain regions (83). A clinical trial indicated that the yoga and meditation was related to significantly reduced depression symptoms, as well as decreased levels of IL-6 (inflammatory cytokines) and increased the activity of telomerase (an enzyme responsible for the length of telomeres, implicated in chronic inflammation) in patients with major depressive disorder (84). In addition, a mindfulness meditation technique was found to reverse many physiological abnormalities due to cytokine- and stress-mediated depression (85).

## 5. Conclusion

Using CAM in the management of depression, as a stand-alone mental illness or a common complication in cancer survivors, has gained increasing attention over the past three decades, and especially since 2002. Mind–body techniques (i.e., yoga and meditation), acupuncture and mindfulness-based intervention are growing research hotspots or emerging trending topics. Given evidence-based results were preferred, research methods such as systematic review and/or meta-analysis were widely used. In addition to appraising the efficacy and safety of CAM therapies, the promising research directions might concentrate on clarifying the possible mechanisms of action underlying CAM therapies in attenuating depression in terms of modulation of psychological stress and neural inflammation. Although countries with mature CAM healthcare systems, such as China, South Korea, and Iran, are enthusiastic about the current research theme, the USA maintained the leading position in this research field published in English. For higher quality research outputs, more intimate cross-country/regional research collaborations are required.

## Data availability statement

The original contributions presented in the study are included in the article/supplementary material, further inquiries can be directed to the corresponding authors.

## Author contributions

F-YZ: Conceptualization, Formal analysis, Writing – original draft, Funding acquisition, Investigation. PX: Methodology, Software, Writing – original draft. ZZ: Supervision, Writing – review & editing. RC: Supervision, Writing – review & editing. YX: Writing – review & editing, Data curation. L-PY: Writing – review & editing, Investigation. H-RW: Funding acquisition, Writing – review & editing. Y-MW: Funding acquisition, Writing – review & editing. Y-XL: Resources, Writing – review & editing. C-YL: Validation, Writing – review & editing. W-JZ: Funding acquisition, Investigation, Writing – review & editing. Q-QF: Visualization, Methodology, Software, Writing – review & editing. GK: Project administration, Writing – review & editing.
