# Supplementary material for: Managing depression with complementary and alternative medicine therapies: a scientometric analysis and visualization of research activities
Source: Front Psychiatry. 2023 Nov 15;14:1288346. doi: 10.3389/fpsyt.2023.1288346 (PMC10684695; doi:10.3389/fpsyt.2023.1288346)
Supplement: Supplementary file 1 [file Data_Sheet_1.pdf]

# Appendices

**Appendix 1 Categories of CAM modalities**

| **Nutritional therapies** | **Psychological therapies** | **Physical therapies** | **Combinations such as psychological and physical or psychological and nutritional therapies** | **Other Complementary Health Approaches** |
|---|---|---|---|---|
| ● Special diets<br>● Dietary supplement<br>● Vitamins and minerals<br>● Herbs<br>● Probiotics<br>● Microbial-based therapies | ● Meditation<br>● Hypnosis<br>● Music therapies<br>● Relaxation therapies (e.g., breathing exercises, guided imagery)<br>● spiritual practice<br>● Mindfulness-based stress reduction<br>● Trager psychophysical integration | ● Acupuncture<br>● Massage<br>● Spinal manipulation<br>● Chiropractic manipulation<br>● Osteopathic manipulation | ● Yoga<br>● Tai-chi<br>● Dance therapies<br>● Some forms of art therapy<br>● Mindful eating<br>● Qi-gong<br>● Pilates | ● Traditional healers<br>● Ayurvedic medicine<br>● Traditional Chinese medicine<br>● Homeopathy<br>● Naturopathy<br>● Functional medicine |

*Notes:* This classification criteria were proposed by the U.S. National Center for Complementary and Integrative Health (https://www.nccih.nih.gov/health/complementary-alternative-or-integrative-health-whats-in-a-name)

**Appendix 2 List of search terms**

| Condition | Context |
|---|---|
| “depression” | “complementary and alternative medicine” |
| “depressive disorder” | “complementary medicine” |
| “depressive episode” | “complementary therapy” |
| | “alternative medicine” |
| | “alternative therapy” |
| | “natural medicine” |
| | “natural therapy” |
| | “naturopathy” |
| | “integrative medicine” |
| | “integrative therapy” |

**Appendix 3 Search strategy for Web of Science Core Collection**

| # | Search Query | Results |
|---|---|---|
| #1 | TS=("depressive disorder") | 49,117 |
| #2 | TS=(depression) | 606,959 |
| #3 | TS=("depressive episode") | 5,982 |
| #4 | #3 OR #2 OR #1 | 624,148 |
| #5 | TS=("complementary and alternative medicine") | 7,680 |
| #6 | TS=("complementary medicine") | 5,534 |
| #7 | TS=("complementary therapy") | 2,141 |
| #8 | TS=("alternative medicine") | 17,014 |
| #9 | TS=("alternative therapy") | 5,602 |
| #10 | TS=("natural medicine") | 979 |
| #11 | TS=("natural therapy") | 198 |
| #12 | TS=("naturopathy") | 473 |
| #13 | TS=("integrative medicine") | 2,784 |
| #14 | TS=("integrative therapy") | 329 |
| #15 | #5 OR #6 OR #7 OR #8 OR #9 OR #10 OR #11 OR #12 OR #13 OR #14 | 30,854 |
| #16 | #15 AND #4 | 1,822 |
| #17 | PY=(1993-2022) | 71,050,371 |
| #18 | #17 AND #16 | 1814 |
| #19 | #17 AND #16 and English (Languages) | 1766 |
| #20 | #17 AND #16 and English (Languages) and Article or Review Article (Document Types) | 1,710 |

## Appendix 4 The top ten countries/regions with the highest productivity

| Rank | Country/Region | *Np* | % of (1710) | *SCP* | *MCP* | *Nc* |
|---|---|---|---|---|---|---|
| 1 | USA | 574 | 33.5 | 519 | 55 | 24,205 |
| 2 | China | 219 | 12.8 | 194 | 25 | 3,114 |
| 3 | Australia | 117 | 6.8 | 85 | 32 | 1,970 |
| 4 | UK | 91 | 5.3 | 76 | 15 | 3,637 |
| 5 | Germany | 89 | 5.2 | 61 | 28 | 1,899 |
| 6 | Canada | 59 | 3.5 | 47 | 12 | 1,311 |
| 7 | Iran | 59 | 3.5 | 54 | 5 | 659 |
| 8 | Korea | 58 | 3.4 | 51 | 7 | 721 |
| 9 | Brazil | 38 | 2.2 | 32 | 6 | 1,211 |
| 10 | Italy | 38 | 2.2 | 29 | 9 | 850 |

***Abbreviations***:

*Np*, number of publications; *Nc*, number of citations; *MCP*, multiple country publications; *SCP*, single country publications.

## Appendix 5 Institutions with the highest productivity (>10 papers)

| Rank | Institution (Country) | *Np* | *Nc* |
|---|---|---|---|
| 1 | Harvard University (USA) | 21 | 9,326 |
| 2 | University of Duisburg-Essen (Germany) | 18 | 537 |
| 3 | Chengdu University of Traditional Chinese Medicine (China) | 18 | 145 |
| 4 | University of Hong Kong (China) | 15 | 515 |
| 5 | University of Technology Sydney (Australia) | 12 | 408 |
| 6 | Western Sydney University (Australia) | 12 | 147 |
| 7 | Northwestern University (USA) | 11 | 394 |

| | | | |
|---|---|---|---|
| 8 | University of Arizona (USA) | 11 | 756 |
| 9 | University of Exeter (UK) | 11 | 1,087 |
| 10 | University of Melbourne (Australia) | 11 | 484 |
| 11 | University of California, Los Angeles (USA) | 10 | 3,312 |

***Abbreviations***:

*Np*, number of publications; *Nc*, number of citations.

## Appendix 6 The top ten most-published journals

| **Rank** | **Journal** | ***Np*** | ***h*_index** | ***g*_index** | ***m*_index** | ***IF* (2022-2023)** | ***JCR* (2022-2023)** | ***Nc*** |
|---|---|---|---|---|---|---|---|---|
| 1 | Journal of Alternative and Complementary Medicine | 61 | 19 | 31 | 0.731 | 2.6 | Q3 | 1,193 |
| 2 | Complementary Therapies in Medicine | 41 | 15 | 21 | 0.882 | 3.6 | Q2 | 533 |
| 3 | Evidence-Based Complementary and Alternative Medicine | 40 | 18 | 30 | 0.9 | N/A | N/A | 957 |
| 4 | Medicine | 33 | 5 | 11 | 0.625 | 1.6 | Q3 | 143 |
| 5 | BMJ Open | 32 | 8 | 12 | 0.727 | 2.9 | Q2 | 168 |
| 6 | Complementary Therapies in Clinical Practice | 30 | 18 | 30 | 0.947 | 3.0 | Q2 | 1,321 |
| 7 | Journal of Affective Disorders | 30 | 11 | 16 | 1 | 6.6 | Q1 | 303 |
| 8 | BMC Complementary Medicine and Therapies | 24 | 14 | 20 | 0.875 | 3.9 | Q2 | 440 |
| 9 | Integrative Cancer Therapies | 23 | 13 | 23 | 0.65 | 2.9 | Q2 | 541 |
| 10 | Supportive Care in Cancer | 23 | 11 | 23 | 0.647 | 3.1 | Q1 | 556 |

***Abbreviations***:

*IF*, Impact Factor; *JCR*, Journal Citation Reports; *Np*, number of publications; *Nc*, number of citations.

**Appendix 7 Trend topics in research regarding CAM therapies for depression over the years**

| Category | Trend topics term | Frequency |
|---|---|---|
| CAM therapies/products | *St Johns Wort* | 45 |
| | Mindfulness | 33 |
| | *Hypericum perforatum* | 22 |
| Mechanism of action | Inflammation | 14 |
| | Psychological stress | 7 |
| Research tools | Depression scale | 23 |
| | Rating scale | 8 |
| Research methods | Meta-analysis | 88 |
| | National survey | 21 |
| | Clinical trials | 20 |
| Disease | Anxiety | 190 |
| | Cancer | 21 |
